# Schizophrenia stigma in mental health professionals and associated factors: A systematic review

**DOI:** 10.1192/j.eurpsy.2022.1580

**Published:** 2022-09-01

**Authors:** K.-M. Valery, A. Prouteau

**Affiliations:** University of Bordeaux, Psychology, Bordeaux, France

**Keywords:** mental health professionals, schizophrénia, stigmatization

## Abstract

**Introduction:**

The consequences of schizophrenia stigma are numerous and highly damaging to individuals, their families, the health care system and society. Mental health professionals (MHP) are considered to be one of the main sources of stigmatization.

**Objectives:**

To identify the characteristics of MHP stigma in schizophrenia in comparison with other psychiatric disorders, the specificities of MHP compared with other social groups, and associated factors.

**Methods:**

Following PRISMA guidelines, we systematically searched multiple electronic databases for articles: (i) reporting original data published in English in peer-reviewed journals, (ii) reporting quantitative data with statistical analysis, (iii) assessing stigma in a broad sense, and (iv) including samples composed only of MHP.

**Results:**

A total of 38 articles published from 1999 to 2019 and involving 10926 MHP fulfilled our inclusion criteria. Studies showed that schizophrenia is the most stigmatized mental illnesses in MHP, despite recent results suggesting that borderline personality disorder and substance abuse may be more stigmatized. In comparison with other social groups, MHP reported less dangerousness beliefs and more positive beliefs regarding pharmacological treatment. Nevertheless, results were less consistent regarding prognosis and desire for social distance. Age, education level, type of mental health profession, or length of practice were associated factors that showed inconsistent relations with stigma. Work setting and biological causal beliefs were more clearly associated with MHP stigma.

**Conclusions:**

These findings provide strong support for the need to conduct specific research on schizophrenia stigma in MHP and the importance of controlling for several variables to identify predictors of stigma.

**Disclosure:**

No significant relationships.

